# The relationship between smoking and cognitive decline: a 4‐year cohort study in rural Xi 'an

**DOI:** 10.1002/alz70860_098740

**Published:** 2025-12-23

**Authors:** Jie Liu, Liangjun Dang, Shan Wei, Ling Gao, Suhang Shang, Xiaojuan Guo, Qiumin Qu, Jin Wang

**Affiliations:** ^1^ The First Affiliated Hospital of Xi'an Jiaotong University, Xi’an, Shaanxi, China; ^2^ The First Affiliated Hospital of Xi'an Jiaotong University, Xi'an, Shaanxi, China; ^3^ The First Affiliated Hospital of Xi’an Jiaotong University, Xi'an, Shaanxi, China; ^4^ The First Affiliated Hospital of Xi'an Jiaotong University, Xi’an, China; ^5^ The First Affiliated Hospital of Xi’an Jiaotong University, Xi’an, Shaanxi, China

## Abstract

**Background:**

Smoking is an important risk factor for cognitive impairment, but the relationship between smoking and cognitive decline remains unclear. This study examined the association between smoking and cognitive decline through a cohort in rural Xi 'an.

**Method:**

The data were collected from the cognitive impairment cohort of middle‐aged and elderly people in rural areas of Xi 'an. This study took the baseline cognitively normal population of this cohort as the research object. According to the survey results in 2018, the population was divided into three groups according to smoking status: smoking, quitting and non‐smoking. Cognitive function was assessed by Mini‐Mental State Examination (MMSE).ΔMMSE (2014 rating ‐2018 rating)≥2 was defined as cognitive decline, and ΔMMSE <2 was defined as cognitive stability. Binary Logistic regression model and stratified analysis were used to analyze the relationship between smoking and cognitive decline and the influence of age on the relationship.

**Result:**

A total of 1289 subjects were included in this study. According to smoking status in 2018, they were divided into non‐smokers (910, 70.6%), smokers (335, 26.0%) and ex‐smokers (44, 3.4%). In the total population, there was no significant difference in the incidence of cognitive decline among non‐smokers, smokers and ex‐smokers (17.3% vs. 16.1% vs. 15.9%, *p* = 0.880). When stratified by age, there was no significant difference in the incidence of cognitive decline among the three groups in the subgroup of age < 65 years (16.5% vs. 13.2% vs. 12.1%, *p* = 0.365). After adjusting for multiple factors, compared with the non‐smoking group, there was no significant difference in the risk of cognitive decline of the smokers (*p* = 0.859) and the ex‐smokers (*p* = 0.976). In the subgroup of age ≥65 years, there was no significant difference in the incidence of cognitive decline among non‐smokers, smokers and ex‐smokers (20.8% vs. 30.9% vs. 27.3%, *p* = 0.303). After adjusting for confounding factors, smokers had a significantly higher risk of cognitive decline than non‐smokers (OR = 14.139; 95%CI: 1.541‐129.705; *p* = 0.019).

**Conclusion:**

Smoking is positively associated with cognitive decline in the elderly, suggesting that smoking may accelerate cognitive decline in the elderly.

